# Relative and contextual contribution of different sources to the composition and abundance of indoor air bacteria in residences

**DOI:** 10.1186/s40168-015-0128-z

**Published:** 2015-12-10

**Authors:** Marzia Miletto, Steven E. Lindow

**Affiliations:** Plant & Microbial Biology, University of California Berkeley, 331 Koshland Hall, Berkeley, CA 94720 USA

**Keywords:** Air microbiology, Source-sink dynamics, Home microbiome, Bioaerosols, Microbial biogeography

## Abstract

**Background:**

The study of the microbial communities in the built environment is of critical importance as humans spend the majority of their time indoors. While the microorganisms in living spaces, especially those in the air, can impact health and well-being, little is known of their identity and the processes that determine their assembly. We investigated the source-sink relationships of airborne bacteria in 29 homes in the San Francisco Bay Area. Samples taken in the sites expected to be source habitats for indoor air microbes were analyzed by 16S rRNA-based pyrosequencing and quantitative PCR. The community composition was related to the characteristics of the household collected at the time of sampling, including the number of residents and pets, activity levels, frequency of cooking and vacuum cleaning, extent of natural ventilation, and abundance and type of vegetation surrounding the building.

**Results:**

Indoor air harbored a diverse bacterial community dominated by *Diaphorobacter* sp*., Propionibacterium* sp*., Sphingomonas* sp*.,* and *Alicyclobacillus* sp*.* Source-sink analysis suggested that outdoor air was the primary source of indoor air microbes in most homes. Bacterial phylogenetic diversity and relative abundance in indoor air did not differ statistically from that in outdoor air. Moreover, the abundance of bacteria in outdoor air was positively correlated with that in indoor air, as would be expected if outdoor air was the main contributor to the bacterial community in indoor bioaerosols. The number of residents, presence of pets, and local tap water also influenced the diversity and size of indoor air microbes. The bacterial load in air increased with the number of residents, activity, and frequency of natural ventilation, and the proportion of bacteria putatively derived from skin increased with the number of residents. Vacuum cleaning increased the signature of pet- and floor-derived bacteria in indoor air, while the frequency of natural ventilation decreased the relative abundance of tap water-derived microorganisms in air.

**Conclusions:**

Indoor air in residences harbors a diverse bacterial community originating from both outdoor and indoor sources and is strongly influenced by household characteristics.

**Electronic supplementary material:**

The online version of this article (doi:10.1186/s40168-015-0128-z) contains supplementary material, which is available to authorized users.

## Background

The study of the indoor microbiome (the microbial communities of the built environment) is of critical importance since humans spend the majority of their time indoors and thus regularly encounter microbes in this habitat. Microbes, including those present in indoor spaces, impact human health and well-being [1, 2]. While initial research in the field of the microbiology of the built environment has mainly focused on microbes of clinical importance, there is growing evidence that a wide variety of microbial taxa are present in the air and on surfaces within buildings. Interrogations of microbial communities have shown that microbes are both diverse and ubiquitous indoors. A diversity of bacteria is found on every surface, especially in kitchens and bathrooms where the environmental conditions are particularly suitable for their immigration and possibly also survival and growth. Bacteria have also been described on pets, indoor plants, foodstuffs, and tap water [3–12]. Bacteria colonize humans, and humans can function as microbial vectors shaping the microbiome of indoor surfaces with which they come in contact [13, 14]. Moreover, dust that has settled on floors and/or carpeting is rich in microbes and, as a complex mixture of inorganic and organic particles, probably represents an integrative record of microbial biodiversity in occupied spaces [15]. Despite demonstrated microbial ubiquity, various microbial taxa exhibit biogeography patterns indoors as well as outdoors [10, 16, 17], suggesting that they are subject to limitations on dispersal and are derived from local sources. In addition, the variation in environmental conditions within buildings (such as humidity, temperature, and availability of nutrients), the intensity or legacy of human usage (e.g., cleaning patterns), and the variation in possible source habitats (e.g., human numbers and features, pets, plants, food, and tap water) are likely to shape the indoor microbiome creating microhabitats colonized by distinct microbial communities.

Air represents a vehicle for movement of microbes from one habitat to another. From indoor sites, microorganisms can directly enter the aerosol phase, such as through the shedding of bacterial-colonized skin cells [18] or the aerosolization of saliva and tap water, and indirectly through the resuspension of settled dust. Irrespective of the means by which they enter the air, they likely represent also an important component of the microbes to which we are exposed [19] given that humans inhale about 10–25 m^3^ of air a day [20]. It would be expected that the contribution of various sources of bacteria to the composition and abundance of indoor air bacteria would be directly related to both their numbers in a given source habitat and the ease by which they enter the air. For example, the relative abundance of human-associated microbes in indoor air increases with the number of residents [21, 22]. Other factors that might influence the contribution of different sources of microbes to the air include human activity levels as well as the frequency of cooking, vacuum cleaning, and showering [23–25]. While there have been many studies of the microbial communities present on humans and the many surfaces within buildings with which they might interact, few studies have attempted to study the relative importance of these various sources on the composition of indoor air, and thus to address issues such as the efficiency of immigration of microbes into the air within buildings. This will be the focus of our study.

Since homes constitute a barrier separating living spaces from the outside world, the degree of exchange between the outdoor microbiome and the indoor microbiome is central to understanding source-sink relationships for indoor air bacterial communities. Outdoor air has been shown to contribute to the indoor air fungal microbiome [26]. Given that microbial communities in outdoor air are both abundant and subject to substantial biogeographic variation [27, 28], it seems likely that they could contribute to the variation in composition or abundance of microbes in indoor air, although this has not been well studied. The biogeographical patterns of airborne bacteria, in turn, are likely driven by spatial patterns of vegetation since the large populations of bacteria on the leaf surface are probably substantial contributors to the air microflora outdoors [29]. It would be expected that the mode of ventilation of a building would influence the composition of airborne bacteria communities indoors. In support of this concept, the phylogenetic diversity of indoor air was found to be lower than that outdoors while the proportion of microorganisms likely originating from indoor sources is higher in indoor air than outdoor air, especially in mechanically ventilated rooms rather than in window-ventilated rooms [30]. While there is a growing number of studies which have addressed the airborne microflora within the built environment, most of these studies have studied institutional buildings such as classrooms or other public spaces such as commercial centers and transit systems which often harbor large number of humans [31]. Furthermore, the study sites are often subject to extensive mechanical ventilation associated with heating and air conditioning systems, which would be expected to strongly impact the abundance and perhaps the identity of microbes found in the air at such sites. Unfortunately, few studies have been made of the airborne microflora in residences which would typically (1) lack the extensive air handling systems present in institutional or commercial buildings, (2) harbor a much lower spatial density of occupants, and which (3) might exhibit airborne communities that are likely distinctive due to their location and the activity of the human occupants.

To better understand the relative importance of various sources of bacteria on the microbial composition of indoor air in residences and thus determine the extent of idiosyncrasy of the microbiome within buildings, we performed an intensive analysis of 29 homes located in the San Francisco Bay Area using 16S rRNA-based pyrosequencing and quantitative PCR. In each residence, samples were collected from indoor air and from a variety of indoor and outdoor sites expected to be likely sources of airborne microbes (kitchen countertops, refrigerator shelves, showerheads, toilet bowls, bathtub tiles, floors, carpeting, residents’ skin, residents’ saliva, pets, tap water, doorsteps, and outdoor air). Considering indoor air to be a “sink” populated by various indoor and outdoor sources, special attention was placed on the role and magnitude of immigration of microbes from outdoor air to the interior of residences; these two air parcels were sampled simultaneously immediately prior to sampling all other sites, and their microbial composition compared at a given residence. Information on the characteristics of the household was collected at the time of sampling including the following: number of residents, activity level, frequency of cooking, frequency of vacuum cleaning, number and type of pets, frequency of natural ventilation, surrounding vegetation coverage, time of sampling, sky cloud coverage, wind, air temperature, and air humidity. While outdoor air was frequently found to be a major source of bacteria found within indoor air, the relative contribution of this and other sources to the indoor air microbiome was strongly influenced by household characteristics and the behavior of the residents.

## Results and discussion

### Microbial community composition in indoor air

**Fig. 1:**
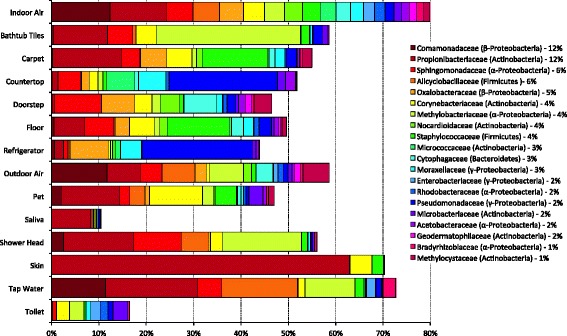
Relative abundance of bacterial communities in indoor air compared to that in potential source environments. *Bars* correspond to the median percentage in the dataset. The percentage for each taxon in indoor air is noted adjacent to the taxon name. Only the 20 most abundant (ca. 80 % of the total sequences recovered) family-level taxa in indoor air are shown. A complete taxonomic breakdown by genera is available in Additional file 1: Table S1

A total of 374 genus-level operational taxonomic units (OTUs) were identified in this study. Approximately 80 % of the sequences found in indoor air samples belong to one of four phyla: Proteobacteria (41 %), Actinobacteria (27 %), Firmicutes (9 %), and Bacteroidetes (3 %) (Fig. 1 and Additional file 1: Table S1). The most represented OTUs we found in indoor air samples are taxonomically related with microorganisms that are ubiquitous in nature and previously encountered in soil, sediments, water and in association with plants, including *Diaphorobacter* (10 % in indoor air), *Alicyclobacillus* (6 %), *Methylobacterium* (4 %), *Sphingomonas* (4 %), *Hymenobacter* (2 %), *Pseudomonas* (2 %), and *Roseomonas* (1 %) (Additional file 2: Table S2 and references therein).

Sequences affiliated with *Propionibacterium* (12 % in indoor air), representative genus of the family *Propionibacteriaceae*, were in the highest proportion in outdoor air, skin, pets, carpet, bathtub tiles, and tap water samples. A similar occurrence in the source environments analyzed was observed for *Corynebacterium* (4 %) and *Staphylococcus* (4 %). These microorganisms typically colonize the skin of humans and other organisms, as well as *Acinetobacter* (2 %) and *Kocuria* (1 %) that, in contrast with *Propionibacterium*, *Corynebacterium*, and *Staphylococcus*, are mainly found on kitchen countertops and refrigerator samples (Additional file 2: Table S2).

Several of these genera have been observed in both outdoor and indoor air in previous studies (Additional file 2: Table S2). Air is an inhospitable environment for microbes and little or no growth is expected, although there is a growing evidence that microorganisms may exhibit metabolic activity while in the airborne state [32]. Nevertheless, traits for enhanced survival in air are necessary if viable cells are to be successfully dispersed to other sites, and many of the OTUs we found in air are related to microorganisms that are capable to resist to desiccation and temperature fluctuations, produce spores, are oligotrophic and/or metabolically versatile, or possess mechanisms to protect from cellular damage due to the exposition to electromagnetic radiation (e.g., the production of pigments and efficient DNA repair systems; Additional file 2: Table S2). Even human-associated microbes (skin and gut) can remain viable in air for many hours after dispersal, as shown in a recent study focused on their ecological succession on restroom surfaces [33].

### Microbial community composition in various source environments

**Fig. 2:**
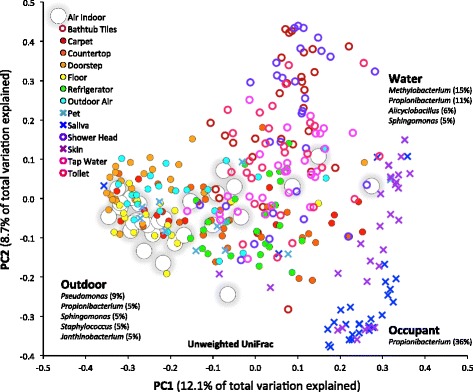
Principal coordinates plot showing the overall variation in bacterial community composition in indoor air and sources. Indoor air bacterial communities in homes (*large open circles*) show various degrees of overlapping with outdoor-related source environments (*closed circles*), indoor-related source environments (*crosses*), and water-related source environments (*open circles*). Differences in the composition of the bacterial communities were quantified using the unweighted UniFrac distance metric and symbols closer together indicate samples with more similar bacterial communities

Bacterial community composition differed substantially between possible source environments. Using qualitative metrics (unweighted UniFrac), sources could be broadly grouped into three clusters (Fig. 2). The first, outdoor-related sources including outdoor air and doorsteps, and also floors, carpets, countertops, and refrigerators (Fig. 2, *closed circles*), were dominated by bacteria belonging to the genera *Pseudomonas*, *Propionibacterium*, *Sphingomonas*, *Staphylococcus*, and *Janthinobacterium* (Additional file 2: Table S2). The second, water-related sources (bathtub tiles, showerheads, tap water, toilets) (Fig. 2, *open circles*) were characterized by a relatively high proportion of bacteria belonging to *Methylobacterium*, *Propionibacterium*, *Alicyclobacillus*, and *Sphingomonas* (Additional file 2: Table S2). The third, resident-related sources such as skin and saliva (Fig. 2, *crosses*) were almost exclusively comprised of *Propionibacterium* sp. (Additional file 2: Table S2). Interestingly, bacterial communities associated with pets clustered with outdoor samples, probably because they have a more diversified composition compared to that of human skin and saliva. Also, given that most of the pets studied here (dogs and cats) spend considerable time outdoors and might be expected to acquire bacteria from the air and other sources outdoors, they might be considered to be “vectors” of such outdoor microbes into the indoor arena, much in the way as humans have been suggested to serve as vectors for such exterior fungi [34]. Importantly, indoor air bacterial communities (Fig. 2, *large open circles*) occupied a central position in this analysis, with various degrees of overlapping with all of the potential source environments, but most prominently with outdoor sources and water sources that likely share the highest number of taxa with indoor air. The separation of the three groups was less evident using quantitative weighted UniFrac metrics (Additional file 3: Figure S1). However, in this analysis, indoor air also had a central position but overlapped equally with outdoor sources and water sources suggesting a similarity of bacterial taxa abundance between the source and sink. In support of this concept, the most represented bacterial OTUs in indoor air were also substantial components of both outdoor air and tap water as the community profiles in Fig. 1 suggest.

**Fig. 3:**
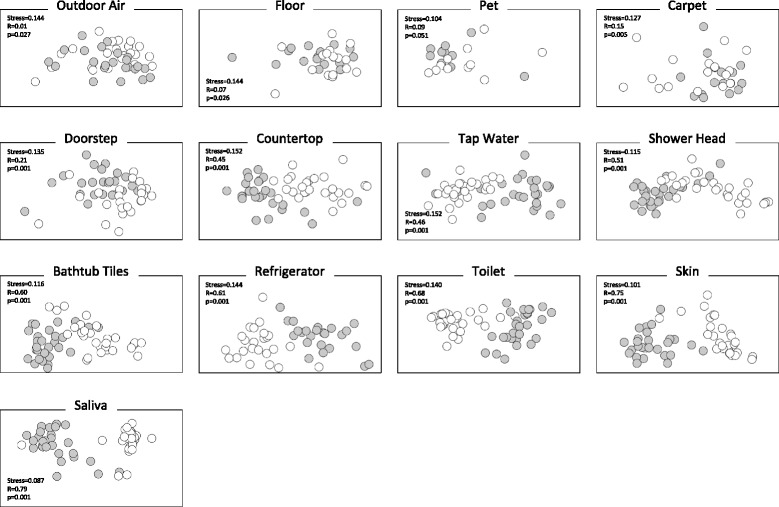
Pairwise unweighted UniFrac distance between indoor air and source environments visualized on a NMDS plot. Indoor air is represented by *open circles*, possible source environments by *closed circles*. Stress as well as ANOSIM *R* and *p* values are indicated (999 permutations). An *R* close to 0 indicates similarity between indoor air microbial communities and the sources; the opposite is true for *R* close to 1

Source-sink pairwise comparisons based on unweighted UniFrac distance metrics (Fig. 3) revealed that indoor air is most similar to that of outdoor air (analysis of similarity (ANOSIM) *R* = 0.01, *p* = 0.027), followed by floors (*R* = 0.07, *p* = 0.026), pets (*R* = 0.09, *p* = 0.051), carpeting (*R* = 0.05, *p* = 0.005), and doorsteps (*R* = 0.21, *p* = 0.001). Countertops, tap water, showerheads, and bathtub tiles exhibited some similarity with indoor air (0.45 < *R* < 0.60, *p* = 0.001), while microbial communities in refrigerators, toilets, skin, and saliva were the most dissimilar to indoor air (0.61 < *R* < 0.79, *p* = 0.001). Similar results were obtained with weighted UniFrac measures (Additional file 3: Figure S2).

### Assignment of origin of indoor air constituents by microbial source tracking

**Fig. 4:**
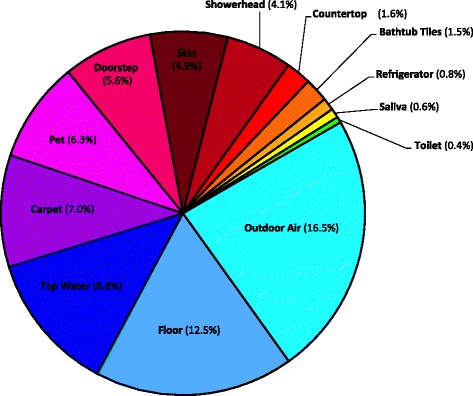
Source environments for indoor air bacterial communities in homes. Sink predictions were determined using SourceTracker. Values represent median contributions across residences of different sources to indoor air. The “unknown source” (sink prediction value of 29.6 %) is not shown. Higher sink prediction values for a source environment indicate a higher proportion of its OTUs in indoor air. The complete set of source-sink predictions values is available in Additional file 4: Table S3

**Fig. 5:**
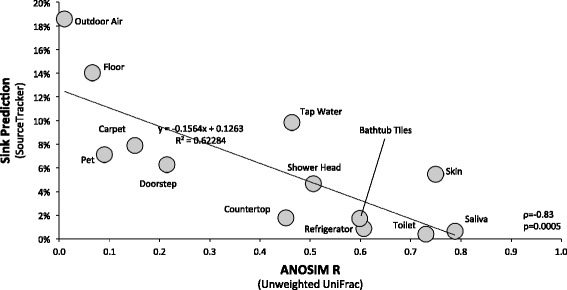
Sink prediction (SourceTracker) in indoor air and unweighted UniFrac phylogenetic distance (ANOSIM *R*) with indoor air for microbial communities in different source environments. Higher sink prediction values for a source environment indicate a higher proportion of its OTUs in indoor air. *R* values close to 0 indicate similarity between indoor air microbial communities and the sources; the opposite is true for *R* values close to 1. The statistical significance (*p*) of the correlation was determined using Spearman’s rank correlation coefficient (*ρ*)

Sink predictions for indoor air microbial communities against different possible source environments across residences were determined using SourceTracker (Additional file 4: Table S3). The results summarized in Fig. 4 suggest that outdoor habitats were the primary sources of bacteria in indoor air in this study. Microbes in outdoor air can be brought into the house via natural ventilation. Likewise, dust deposited on doorsteps can be carried indoors by residents. A link between the bacterial taxa deposited on outdoor surfaces and those found on indoor surfaces, highlighting the direct effect that outdoor microbes can have on the microbial communities living within our homes, has also been shown elsewhere [17]. In our survey, sources related to household residents (bacteria released from human skin and pets) were also among the major contributors of indoor air bacteria (Fig. 4). Recently, human skin was shown to be an important source of bacteria on residential surfaces [10, 13], and the presence of dogs was linked to a more diverse microbial community and higher relative abundances of dog-associated bacterial taxa on surfaces in homes [17]. The floor and carpeting also contributed substantially to bacteria recovered in indoor air (Fig. 4); both environments constitute a reservoir of immigrant bacteria that have been previously deposited there from indoor and outdoor sources, as well as a source of emigrant indoor air microbes. Interestingly, tap water was indicated to be the third most important source of bacteria in indoor air, and unsurprisingly, was similar to that of the showerhead. Both these source environments may present a significant potential exposure to aerosolized microbes, including documented opportunistic pathogens [7]. In our survey, the source environments that contributed little to indoor air were kitchen countertops, bathtub tiles, refrigerators, saliva, and toilets. Source tracking results support those obtained with distance metrics; the SourceTracker prediction of the contribution of a source to the sink (indoor air) is significantly inversely correlated to the magnitude of their ANOSIM *R*, as depicted in Fig. 5 (*ρ* = −0.83, *p* = 0.0005; unweighted UniFrac) and Additional file 3: Figure S3 (*ρ* = −0.81, *p* = 0.0007; weighted UniFrac).

### Outdoor air is the primary source of bacteria in indoor air in these residences

**Fig. 6:**
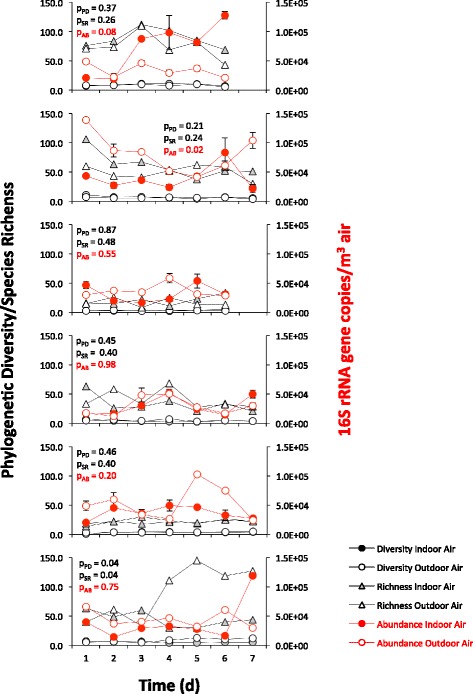
Temporal dynamics of bacterial diversity, bacterial species richness, and bacterial abundance in indoor air and outdoor air. Bacterial diversity (Faith’s phylogenetic diversity) is indicated by *black circles* (indoors: *closed markers*; outdoors: *open markers*). Species richness is indicated by *grey triangles* (indoors: *closed markers*; outdoors: *open markers*). Bacterial abundance in indoor air (*red closed circles*) and outdoor air (*red open circles*) was quantified by counting the 16S rRNA gene copies in a cubic meter of air. The *t* test statistics was used to test the null hypothesis that there is no difference between the bacterial diversity (*p*_PD_), species richness (*p*_SR_), and abundance (*p*_A_) in indoor air compared to outdoor air (a *p* value ≥0.05 confirms the null hypothesis)

As would be expected if outdoor air was a major contributor to the composition of bacteria in indoor air, both bacterial phylogenetic diversity, species richness, and bacterial abundance in indoor and outdoor air changed with time in several residences that were repeatedly sampled over a period of 1 week (Fig. 6). Importantly, however, both diversity and abundance of the indoor and outdoor air samples tended to change in tandem, and neither the abundance nor diversity of the airborne communities in indoor and outdoor air differed statistically from each other over time (Fig. 6). The Bay Area climate is a typical warm-summer Mediterranean climate, characterized by warm dry summers and rainy mild winters. The climate is mild year-round with little seasonal temperature variation: the average monthly temperatures of 22 °C during its warmest month and between 18 and −3 °C (64 to 27 °F) in its coldest month. For this reason, it is common to ventilate rooms by opening windows, and this may have resulted in the relatively large contribution of outdoor air to the composition of the indoor air microbiome. It might be expected that in other regions with more extreme climates, where it is impractical to open windows frequently, indoor sources may contribute a higher proportion of the bacteria to the indoor air and a lesser contribution would be made by outside air. Our results are consistent with a recent study by Meadow at al. [22] that showed the importance of the ventilation strategy in shaping the microbial community inside a university office building; indoor air communities closely tracked outdoor air communities, especially in rooms that were naturally ventilated overnight. It is clear, however, that the indoor air microflora is quite dynamic, with frequent changes in both the abundance and composition associated with changes in both resident activity, ventilation patterns, and apparently also of rather large changes in the bacterial composition of outside air, possibly driven by changes in wind direction and wind speed that might alter the sources of aerosolized bacteria, likely from plants nearby.

**Fig. 7:**
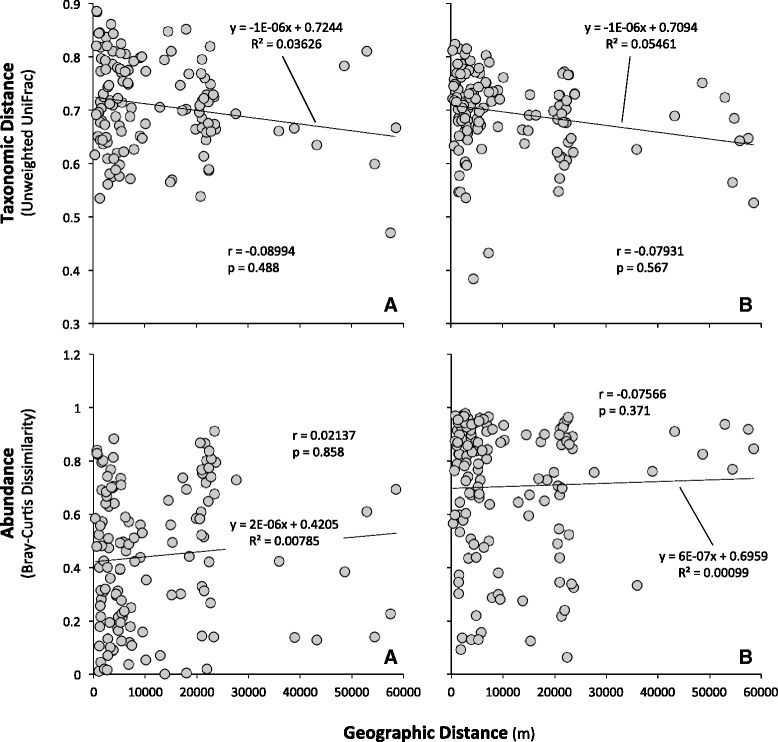
Correlation between the taxonomic distance or abundance and geographic distance for indoor air **(a)** and outdoor air **(b)** bacterial communities. The taxonomic distance was determined using unweighted UniFrac metrics. The distance in abundance data was determined using Bray-Curtis dissimilarity. The statistical significance was determined using the Mantel *r* statistics (999 permutations)

Our study revealed that there was no significant correlation between geographic distance and (1) taxonomic distance for indoor air bacterial communities (Mantel *r* = −0.00035, *p* = 0.996), (2) taxonomic distance for outdoor air bacterial communities (Mantel *r* = −0.05835, *p* = 0.643), and (3) bacterial abundance in indoor and outdoor air (Fig. 7). While a small decrease in taxonomic similarity with increasing geographical distance was observed for outdoor bacteria as has been observed for outdoor fungi [35], this relationship was not statistically significant. It is possible that the sources of outdoor bacteria are quite variable, and assuming that they are dispersal-limited as has been observed for fungi and other bacteria [26], variation in different local sources may have obscured any larger geographical signature that might also have been present. It is tempting to speculate that plant surfaces are a major source of airborne bacteria as suggested in other studies [27, 28]. Given that different plant species would be expected to harbor different epiphytic bacterial populations [29], the considerable variation in plant species distribution in urban settings such as the San Francisco Bay Area might be expected to lead to corresponding variation in the amount and type of bacteria near a given residence.

**Fig. 8:**
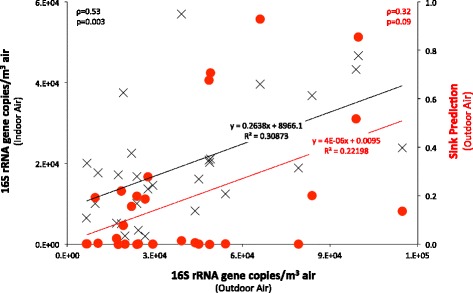
Relationship between the abundance of bacteria in outdoor air and the abundance of bacteria in indoor air (*black markers*) and the sink predictions for outdoor air (*red markers*). Bacterial abundance was estimated counting the 16S rRNA gene copy number in a cubic meter of air. The sink prediction for outdoor air was calculated using SourceTracker. The statistical significance (*p*) of the correlation was determined using Spearman’s rank correlation coefficient (*ρ*)

The abundance of bacteria in outdoor air was positively correlated with the abundance of bacteria in indoor air in a given residence at a given time (*ρ* = 0.53, *p* = 0.003; Fig. 8). Likewise, a positive relationship was observed between the abundance of outdoor bacteria and the proportion of the total community that was predicted deriving from outdoor air using SourceTracker (*ρ* = 0.32, *p* = 0.05). These positive relationships would be expected if outdoor air was a large contributor to the bacterial community in indoor air. That is, the introduction of a parcel of outdoor air containing a given concentration of bacteria would be expected to modulate the concentration of bacteria within the inside air. The introduction of outdoor air with high bacterial concentrations would lead to indoor air also having relatively high bacterial concentrations. Likewise, outside air having relatively high bacterial concentrations would have a larger effect on the composition of bacteria in indoor air, all other factors being constant.

### Effect of the household characteristics on the abundance and diversity of bacteria in air

Correlation analysis between bacterial abundance in indoor air and household characteristics revealed that bacterial load in air significantly (*p* < 0.05) increased with the frequency of natural ventilation (*ρ* = 0.56), number of residents (*ρ* = 0.45), and activity level (*ρ* = 0.40; Table 1). Furthermore, the proportion of bacteria associated with skin was significantly positively correlated with the number of residents within a dwelling (*ρ* = 0.45, *p* < 0.05). Such a relationship would be expected, as an increased number of people within the building as well as the number of hours spent indoors would increase the potential for them to shed skin cells colonized by bacteria and to resuspend microbial cells from the carpet and floor to the indoor air as shown previously [21]. The sink prediction for pets as a source of indoor bacteria was correlated positively with the frequency of vacuum cleaning (*ρ* = 0.46, *p* < 0.05). Given that pets interact closely with carpeting and other flooring, and are a major source of particles that would be found in those habitats, it would be expected that vacuum cleaning would resuspend such particles, leading to the relatively high numbers of pet-associated microbes seen in indoor air in those residences with pets. Similar considerations can be made for the prediction for floors as a source, given that the prominence of sequences associated with floors also increased with the frequency of vacuum cleaning (*ρ* = 0.52, *p* < 0.05). The sink prediction for tap water was negatively associated with the frequency of natural ventilation (*ρ* = −0.40, *p* < 0.05). This relationship might be expected if ventilation conferred by opening of windows caused a loss of bacteria aerosolized from tap water away from the residence.

**Table 1:** Effect of the household characteristics on the abundance and diversity of bacteria in air

	Number of residents	Activity^a^	Cooking^b^	Vacuum cleaning^c^	Number of pets	Natural ventilation^d^	Vegetation^e^
Bacterial abundance	*0.45*	*0.40*	0.36	0.23	0.33	0.56	−0.05
Sink prediction							
Bathtub tiles	0.14	0.03	−0.32	0.08	−0.13	0.33	−0.03
Carpet	−0.02	0.04	0.21	0.06	0.27	0.08	0.09
Counter top	−0.31	−0.29	−0.17	0.06	0.31	0.07	0.10
Doorstep	−0.08	−0.18	0.05	0.05	−0.12	0.29	0.01
Floor	0.04	−0.14	−0.21	*0.52*	0.19	0.09	0.46
Refrigerator	−0.11	0.05	−0.04	0.07	−0.28	−0.10	0.00
Outdoor air	0.10	0.33	0.11	0.01	0.06	−0.28	−0.06
Pet	0.14	−0.02	0.21	*0.46*	0.33	−0.22	0.06
Saliva	0.19	−0.11	−0.04	−0.04	−0.08	−0.39	−0.31
Showerhead	−0.03	−0.08	−0.24	0.21	−0.25	−0.25	−0.34
Skin	*0.47*	−0.16	0.21	−0.22	−0.18	−0.18	−0.01
Tap water	−0.15	−0.30	−0.28	−0.02	−0.15	*−0.40*	−0.36
Toilet	0.08	0.24	−0.02	−0.26	−0.15	0.09	0.26

Values represent the Spearman’s rank correlation coefficient (*ρ*) between bacterial abundance in indoor air and sink prediction in indoor air for various source environments and household characteristics. Correlations in italics are significant at a *p* value <0.05

^a^Daily hours spent indoors

^b^Times per week

^c^Times per month

^d^Daily hours

^e^Percentage of coverage

## Conclusions

Indoor air harbors a diverse collection of bacteria originating from both indoor and outdoor sources. While bacteria are present on many surfaces within residences, they are apparently not efficiently introduced into the air from most of such sites, as the composition of bacteria communities in the air did not resemble that of most inanimate surfaces. In contrast, the escape of bacteria from tap water to interior air appears to be more efficient than from surfaces. While humans themselves and their pets can be substantial sources of bacteria that enter indoor air in residences, their contribution is influenced by the number and activity of the residents. Given that the concentration of bacteria in outdoor air was usually higher than that of indoor air and ventilation such as by opening doors and windows could efficiently replace indoor air with that from external sites, outdoor air was a major contributor of bacteria to the residences studied.

## Methods

### Sites and sample collections

Sampling of 29 single-family homes or individual apartment units in multiunit buildings located in the San Francisco Bay Area, California, took place between April and May 2013. In each residence, surface samples were collected a single time from 12 sites (indoor sources): kitchen countertop, refrigerator shelves, showerhead, toilet bowl, bathtub tiles, floor (tiles, wood, linoleum), carpet (fitted carpeting, rug), residents’ skin (forehead), residents’ saliva, pet (fur, scales, feathers, or designated pet area e.g., cage), tap water, and doorstep. In addition, outdoor air (outdoor source) and indoor air (considered a sink) was simultaneously sampled at each site. These surface sampling sites were present in most residences allowing enough replication for downstream statistical analyses (most source environments, *n* = 29; floor, *n* = 19; carpet, *n* = 17; pet, *n* = 12). These sites were chosen because they were expected to be locations of the highest microbial abundance and/or were considered to be most likely to be aerosolized. For six of the residences, selected based on volunteer availability, indoor and outdoor air samples were collected daily for seven consecutive days. Indoor air and outdoor air were sampled simultaneously prior to collecting any other sample. No residents were at home during aerosol sampling. While sampling was not done at the same time of day for each residence, all samples were collected during daylight hours and soon after the residents had left their homes. It was therefore expected that contributions from resident activities on airborne microflora was proportional to their numbers or behaviors and not strongly influenced by sampling time itself. For indoor sampling, the filtration apparatus was placed in the middle of the living room (central position within each residence) and consisted of a sterile filter cassette equipped with a 0.22-μm cellulose nitrate filter (Fisher Scientific, Pittsburgh, PA, USA) suspended 1 m above the floor by means of a tripod and connected with tubing to a vacuum pump (High Output Vacuum/Pressure Pump; Millipore, Billerica, MA, USA). Outdoor samples were taken simultaneously by a similar method at a site within about 5 m of the entry to each residence. Approximately 1 m^3^ of air was filtered by operating the pump at a constant vacuum/flow rate over a period of 1 h. Filters were sealed and stored frozen at −20 °C until DNA extraction. Tap water (50 mL) was collected after flushing of plumbing for 2 min from a bathroom faucet in a sterile tube and stored on ice until returning to the lab. There, water was immediately filtered through a 0.22 μm cellulose nitrate filter, and the filter was sealed and stored at −20 °C until processing. Surfaces were sampled for 10 s using sterile, cotton-tipped swabs. Immediately after sampling, the tip of the sampling swab was excised directly into a PowerSoil® Bead Tube (PowerSoil®DNA Isolation Kit; MoBio, Solana Beach, CA, USA) and stored on ice until return to the lab. For each household, skin and saliva swabs taken from each resident were pooled to protect volunteer confidentiality. Genomic DNA was extracted from the swabs on the same day of sampling, while air and water samples (filters) were processed at the end of the sampling campaign. Information on the characteristics of the household was collected at the time of sampling including the following: number of residents, activity level (average hours spent at home daily), frequency of cooking (times per week), frequency of vacuum cleaning (times per month), number and type of pets, frequency of natural ventilation (average hours daily), and surrounding vegetation coverage (Additional file 5: Table S4). Additional information included: time of sampling, sky cloud coverage, wind (http://www.wunderground.com), air temperature, and humidity (HOBO T/Rh data logger; Onset Computer Corp., Bourne, MA, USA). The sampling protocol was approved by the University of California Committee for the Protection of Human Subjects (protocol ID #2011-03-2947).

### DNA extraction

Filters were thawed and sliced aseptically in a DNA-free work area, using a razor blade treated by immersion in DNA AWAY™ (Thermo Scientific, Waltham, MA, USA) and ethanol and then flamed prior to use. Filter segments were loaded into a PowerSoil® Bead Tube (MoBio, Solana Beach, CA, USA). Tubes were processed according to the manufacturer’s protocol, following 30 s of beating at maximum speed after the addition of solution C1 to the PowerSoil® Bead Tube. A total of 437 DNA samples were extracted. Extraction controls were processed to exclude the presence of contaminations on reagents (no sample), filters (sterile filter), and swabs (sterile swab).

### Library preparation

Approximately 381 bp from the 16S rRNA hypervariable region V2 was amplified from each DNA sample in triplicates and pooled. Each sample was amplified with a unique barcode to enable multiplexing in the 454 runs. Fusion primers for unidirectional sequencing (Lib-L) were designed from primers UNIV27F (5′-AGAGTTTGATCCTGGCTCAG-3′) and BACT338R (5′-TGCTGCCTCCCGTAGGAGT-3′) according to Roche guidelines. In particular, forward fusion primer included the barcode (MID1-100; Roche Molecular Diagnostics, Pleasanton, CA, USA) and primer BACT338R to obtain a good read of the target region. Negative controls were included in each PCR assays. The reaction mixture contained 5 μL of genomic DNA extract, 0.5 μL of each fusion primer (30 μM), 20 μL of 5 PRIME MasterMix (1×), and 24 μL of PCR quality water. Thermal cycling conditions were the following: 3 min at 94 °C, followed by 30 cycles of 45 s at 94 °C, 30 s at 50 °C, and 1.5 min at 72 °C. The cycling was completed by a final elongation step at 72 °C for 10 min. The post-PCR cleanup was performed with magnetic beads (Agencourt® AMPure® XP PCR Purification System; Agencourt Bioscience Corporation, Beverly, MA, USA). Amplicons were quantified using the Invitrogen Qubit™ dsDNA HS Assay Kit (Invitrogen, Carlsbad, CA, USA) and multiplexed at an equimolar concentration (10 ng/μL). Negative extraction controls did not yield enough amplicons for sequencing. Samples (434) were split in 17 libraries and sequenced on a Roche GS FLX+ System at the University of Illinois (W.M. Keck Center for Comparative and Functional Genomics, University of Illinois, Urbana-Champaign). Information on all the processed samples is detailed in the mapping file (Additional file 6: Table S5).

### Sequence processing

All sequences generated for this study were processed using the default parameters in QIIME [36]. In brief, demultiplexing included a quality filter (minimum quality score of 25), the removal of the reverse primer and any sequence from the end of each read, and a length filter (min = 300 bp, max = 400 bp). After quality control and barcode assignment, the remaining high-quality reads (867,567) from each run were merged in a single fasta file. Sequences were binned into OTUs at a 97 % sequence similarity cutoff using UCLUST [37]. Representative sequences for each OTU were assigned taxonomy with the RDP Classifier [38] and aligned using PyNAST [39] against the Greengenes core set [40]. Chimeric sequences were identified with ChimeraSlayer [41] and removed from the database, as well as singletons, phylotypes classified as chloroplasts and mitochondria, and OTUs present in less than 1 % of the samples. Samples were rarefied to 100 per sample to eliminate potential biases introduced by uneven sampling depth. Samples with fewer than 100 sequences were excluded from taxonomic, alpha-diversity, beta-diversity, and source tracking analyses.

### Data analysis

Data analysis relied on the software QIIME and R [42]. The qualitative (unweighted) and qualitative (weighted) UniFrac metrics were used to determine the phylogenetic distance of the bacterial communities associated with indoor air and the putative sources. Distances were visualized on a nonmetric multidimensional scaling (NMDS) plot, and the statistical significance of similarity between indoor air and sources was analyzed with ANOSIM available in the vegan R package (999 permutations, [43]). The SourceTracker software package [44] was used to determine the potential sources of bacteria in indoor air and their importance in the households sampled. Phylogenetic diversity (Faith’s PD) for indoor and outdoor air was calculated with the pd function in the picante package in R [45]. Mantel test (999 permutations) was used to test the correlation between the taxonomic distance matrix built from indoor and outdoor bacterial community composition data (UniFrac), the geographic distance, and the distance between the households calculated from abundance data of bacteria in indoor and outdoor air using Bray-Curtis dissimilarity metrics. The Spearman’s rank correlation coefficient (*ρ*) was used to measure the strength of the relationship between the sets of data produced in this study. The Student’s *t* test was used to determine if indoor air and outdoor air are significantly different from each other based on Faith’s PD and bacterial abundance.

### Bacterial quantification

Bacterial abundance was determined by qualitative PCR (qPCR) in polypropylene 96-well plates on a 7500 Real-Time PCR System (Applied Biosystems Inc., Foster City, CA, USA). A 16S rRNA fragment of approximately 180 bp was amplified using primers EUB338F (5′-ACTCCTACGGGAGGCAGCAG-3′; [46]) and EUB518R (5′-ATTACCGCGGCTGCTGG-3′; [47]) following a protocol previously described [48]. Briefly, each 25-μL qPCR mixture contained 12.5 μL of Power SYBR green PCR Master Mix (Applied Biosystems Inc., Foster City, CA, USA), 1.5 μL of a 150-nM concentration of each primer, and 9.5 μL of a 1:10 dilution of genomic DNA template. PCR conditions were as follows: 15 min at 95 °C followed by 40 cycles of 1 min at 95 °C, 30 s at 55 °C, and 1 min at 72 °C. Standard curves were constructed with serial dilutions of known amounts of 16S RNA genes amplified with primers EUB338F and EUB518R from environmental genomic DNA. Serial dilutions covered a range of 8 orders of magnitude of template copies per assay (10^2^ to 10^9^). *R*^2^ values ranged from 0.996 to 0.999. The qPCR efficiency (97 to 100 %) was calculated based on the slope of the standard curve. All qPCR assays were run in triplicates. Melting curve analysis of the qPCR products was conducted for each assay to confirm the specificity of the qPCR assays. Target copy numbers for each reaction were calculated from the standard curves assuming an average molar mass of a DNA base pair of 660 g mol^−1^. Correct amplicon size was verified by running aliquots of qPCR on an ethidium bromide-stained 1 % agarose gel. Genomic DNA extracts were tested for PCR-inhibitory substances running qPCR assays on a serial dilution of the template genomic DNA. Templates were normalized to an equal amount of genomic DNA to enable comparison of results.

### Availability of supporting data

The sequence data set supporting the results of this article is available in the FigShare repository [10.6084/m9.figshare.1525083]. All additional files supporting the results of this article are included within the article and its additional files.

## Additional files

Additional file 1: Table S1. OTU table. Genus-level taxonomic composition of bacterial communities associated with indoor air and sources. Columns correspond to samples, rows correspond to OTUs, and values represent the number of times an OTU appears in a particular sample. (xlsx 684 kb) Additional file 2: Table S2. Most abundant bacterial genus-level OTUs in indoor air compared to potential source environments. Values represent the median coverage (%) in the sequence dataset. Only the 20 most abundant (ca. 80 % of the sequences) family-level taxa in indoor air are shown. The representative genus in the OTU is shown [22, 49–68]. (xlsx 18.7 kb) Additional file 3: Figures S1-S4. Figure S1 Principal coordinate plot showing the overall variation in bacterial community composition in indoor air and sources. Indoor air bacterial communities in homes (*large open circles*) show various degrees of overlapping with outdoor-related source environments (*closed circles*), indoor-related source environments (*crosses*), and water-related source environments (*open circles*). Differences in the composition of the bacterial communities were quantified using the weighted UniFrac distance metric and symbols closer together indicate samples with more similar bacterial communities. **Figure S2.** Relationship between weighted and unweighted UniFrac phylogenetic distance (ANOSIM *R*) between microbial communities in indoor air and different source environments. *R* values close to 0 indicate similarity between indoor air microbial communities and the sources; the opposite is true for *R* values close to 1. The statistical significance (*p*) of the correlation was determined using Spearman’s rank correlation coefficient (*ρ*). **Figure S3.** Sink prediction (SourceTracker) in indoor air and weighted UniFrac phylogenetic distance (ANOSIM *R*) with indoor air for microbial communities in different source environments. Higher sink prediction values for a source environment indicate a higher proportion of its OTUs in indoor air. *R* values close to 0 indicate similarity between indoor air microbial communities and the sources; the opposite is true for *R* values close to 1. The statistical significance (*p*) of the correlation was determined using Spearman’s rank correlation coefficient (*ρ*). **Figure S4.** Correlation between the taxonomic distance and geographic distance for indoor air **(a)** and outdoor air **(b)** bacterial communities. The taxonomic distance was determined using weighted UniFrac metrics. The statistical significance was determined using the Mantel *r* statistics (999 permutations). (PDF 281 kb) Additional file 4: Table S3. Sink predictions in indoor air (SourceTracker) for different indoor and outdoor sources. Sink predictions values were determined using SourceTracker. Higher sink prediction values for a source environment indicate a higher proportion of its OTUs in indoor air. (xlsx 43 kb) Additional file 5: Table S4. Metadata associated with all residences sampled in this study. Information on the characteristics of the household was collected at the time of sampling. (xlsx 12.1 kb) Additional file 6: Table S5. Mapping file containing information on all samples analyzed in this study, including the barcode identifying each sample in the sequence data set. This information was used during sequence processing and data analysis, in particular with QIIME, R and SourceTracker. (xlsx 56.6 kb)
