# Supplementary figures and images for: Relative and contextual contribution of different sources to the composition and abundance of indoor air bacteria in residences
Source: Microbiome. 2015 Dec 10;3:61. doi: 10.1186/s40168-015-0128-z (PMC4674937; doi:10.1186/s40168-015-0128-z)

Figure S1

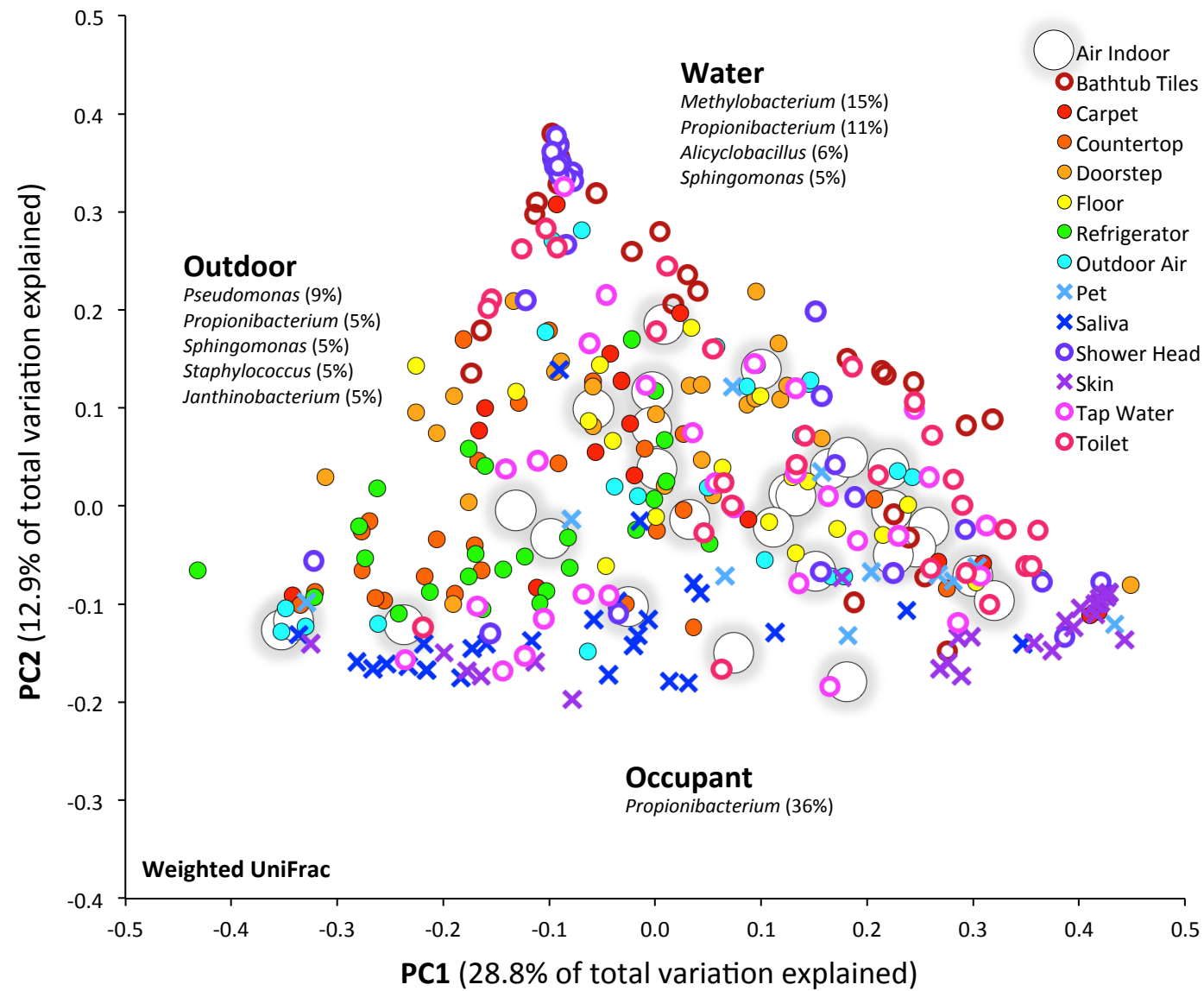

Figure S2

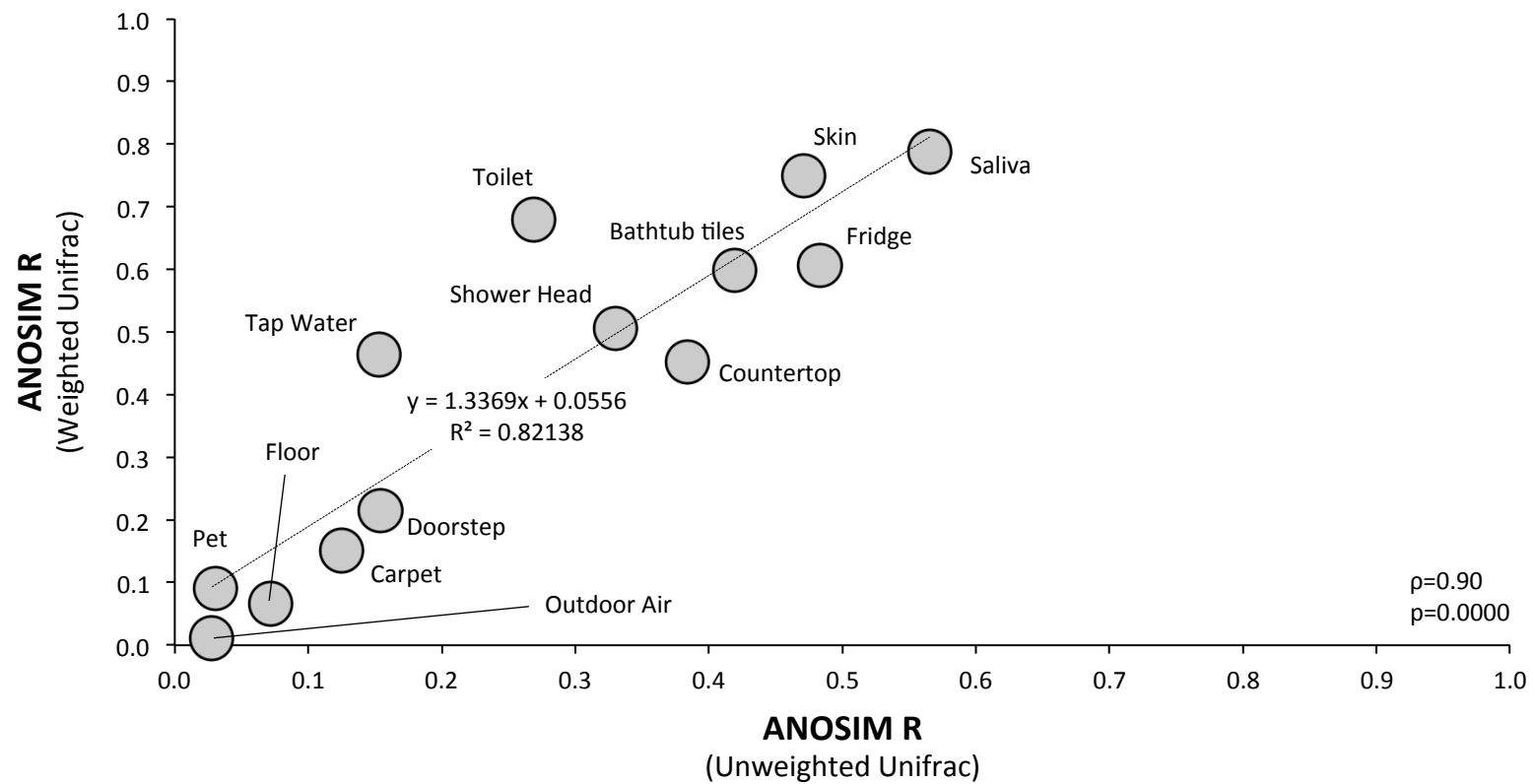

Figure S3

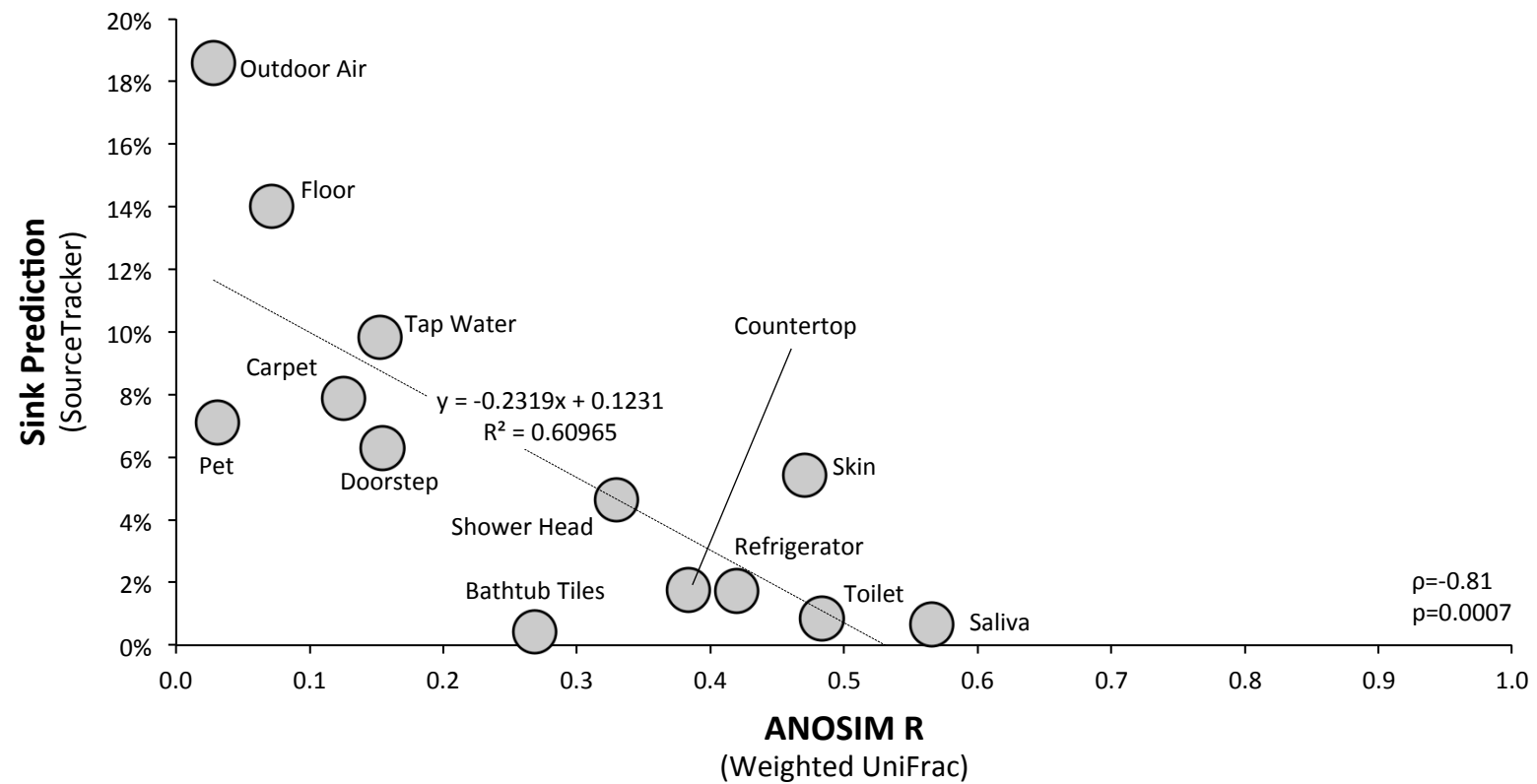

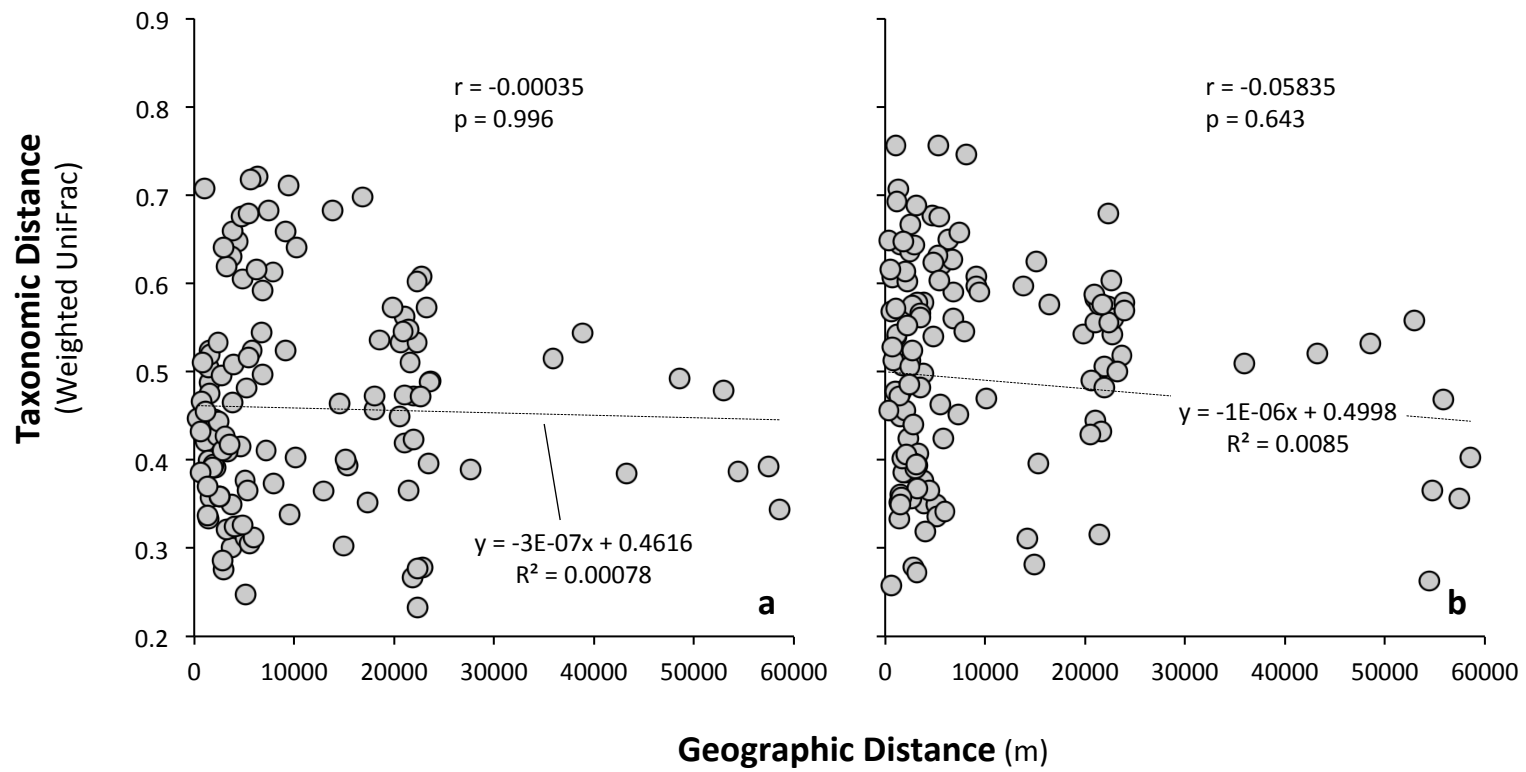

Supplement: Additional file 3: Figures S1-S4. Figure S1 — Principal coordinate plot showing the overall variation in bacterial community composition in indoor air and sources. Indoor air bacterial communities in homes (large open circles) show various degrees of overlapping with outdoor-related source environments (closed circles), indoor-related source environments (crosses), and water-related source environments (open circles). Differences in the composition of the bacterial communities were quantified using the weighted UniFrac distance metric and symbols closer together indicate samples with more similar bacterial communities. Figure S2. Relationship between weighted and unweighted UniFrac phylogenetic distance (ANOSIM R) between microbial communities in indoor air and different source environments. R values close to 0 indicate similarity between indoor air microbial communities and the sources; the opposite is true for R values close to 1. The statistical significance (p) of the correlation was determined using Spearman’s rank correlation coefficient (ρ). Figure S3. Sink prediction (SourceTracker) in indoor air and weighted UniFrac phylogenetic distance (ANOSIM R) with indoor air for microbial communities in different source environments. Higher sink prediction values for a source environment indicate a higher proportion of its OTUs in indoor air. R values close to 0 indicate similarity between indoor air microbial communities and the sources; the opposite is true for R values close to 1. The statistical significance (p) of the correlation was determined using Spearman’s rank correlation coefficient (ρ). Figure S4. Correlation between the taxonomic distance and geographic distance for indoor air (a) and outdoor air (b) bacterial communities. The taxonomic distance was determined using weighted UniFrac metrics. The statistical significance was determined using the Mantel r statistics (999 permutations). (PDF 281 kb) [file 40168_2015_128_MOESM3_ESM.pdf]
